# Comparative Genomic Analysis of the *GRF* Genes in Chinese Pear (*Pyrus bretschneideri Rehd*), Poplar (*Populous*), Grape (*Vitis vinifera*), *Arabidopsis* and Rice (*Oryza sativa*)

**DOI:** 10.3389/fpls.2016.01750

**Published:** 2016-11-24

**Authors:** Yunpeng Cao, Yahui Han, Qing Jin, Yi Lin, Yongping Cai

**Affiliations:** ^1^School of Life Sciences, Anhui Agricultural UniversityHefei, China; ^2^State Key Laboratory of Tea Plant Biology and Utilization, Anhui Agricultural UniversityHefei, China

**Keywords:** GRF, microsynteny, gene structure, molecular evolution, gene duplication

## Abstract

Growth-regulating factors (GRFs) are plant-specific transcription factors that have important functions in regulating plant growth and development. Previous studies on GRF family members focused either on a single or a small set of genes. Here, a comparative genomic analysis of the *GRF* gene family was performed in poplar (a model tree species), *Arabidopsis* (a model plant for annual herbaceous dicots), grape (one model plant for perennial dicots), rice (a model plant for monocots) and Chinese pear (one of the economical fruit crops). In total, 58 *GRF* genes were identified, 12 genes in rice (*Oryza sativa*), 8 genes in grape (*Vitis vinifera*), 9 genes in *Arabidopsis thaliana*, 19 genes in poplar (*Populus trichocarpa*) and 10 genes in Chinese pear (*Pyrus bretschneideri*). The *GRF* genes were divided into five subfamilies based on the phylogenetic analysis, which was supported by their structural analysis. Furthermore, microsynteny analysis indicated that highly conserved regions of microsynteny were identified in all of the five species tested. And Ka/Ks analysis revealed that purifying selection plays an important role in the maintenance of *GRF* genes. Our results provide basic information on *GRF* genes in five plant species and lay the foundation for future research on the functions of these genes.

## Introduction

Growth-regulating factors (GRFs) are plant-specific proteins. The first identified GRF, was rice *OsGRF1* (van der Knaap et al., 2000). Subsequent studies found that *GRF* genes played a critical role in the regulation of plant growth and development (Kim et al., 2003; Horiguchi et al., 2005; Kuijt et al., 2014; Liang et al., 2014; Vercruyssen et al., 2015). In recent years, with the sequencing of tens of plant genomes, many *GRF* genes have been isolated and identified. The N-terminal of *Arabidopsis* GRF9 protein and Chinese cabbage GRF12 contain two WRC (Trp, Arg, Cys) structure domains (Kim et al., 2003; Wang et al., 2014), whereas the N-terminal of GRF proteins have one WRC and one QLQ (Gln, Leu, Gln) structure domain in the species studied (van der Knaap et al., 2000; Kim et al., 2003; Choi et al., 2004). The QLQ structure domain is similar to the N-terminal of SWI2/SNF2 in yeast, which could combine with SNF11 to form the chromatin remodeling complex (Treich et al., 1995). In addition, this structure domain could interact with the SNH structure domain in GIF (GRF-interacting factor) to form a functional complex to perform a transcriptional activation function (Kim and Kende, 2004). The WRC domain consists of one functional nuclear localization signal and one DNA binding motif (zinc finger structure), which is mainly involved in DNA binding. Moreover, the C-terminals of some GRF proteins also include the TQL (Thr, Gln, Leu), GGPL (Gly, Gly, Pro, Leu) and FFD structure domains (Kim et al., 2003; Choi et al., 2004; Zhang et al., 2008; Wang et al., 2014). The transcriptional expression of the *GRF* gene was found to be regulated by GA. For instance, after celery cabbage leaves were treated with GA3, the transcriptional expression levels of the *BrGRF5, BrGRF8, BrGRF9, BrGRF11, BrGRF12, BrGRF13, BrGRF15*, and *BrGRF16* genes were increased by more than fivefolds and those of *BrGRF2, BrGRF4, BrGRF7* were increased by more than 2–5 folds compared with controls (Wang et al., 2014). Moreover, miR396 is also involved in the regulation of *GRF* gene expression. The ath-miR396a gene of *Arabidopsis thaliana* was over-expressed and caused decreased *GRF* gene transcription levels (*FG137771, FG165999, FG194560*) in tobacco. Moreover, the petal, stamen and carpel of transgenic plants increased and fertility was reduced (Yang et al., 2009).

Although the *GRF* gene family has been reported in plants such as Chinese cabbage (Wang et al., 2014), *Cucurbitaceae* (Baloglu, 2014), *Brachypodium distachyon* (Filiz et al., 2014), and *Zea mays* (Zhang et al., 2008), both the mechanism of *GRF* gene expansion and specific evolutionary relationships remain elusive. Comparative genomic studies in plants would clarify the genome evolution by microsynteny analysis across different species. In our study, we analyzed the *GRF* genes from five flowering plant species including pear (*Pyrus bretschneideri*), poplar (*Populus trichocarpa*), *Arabidopsis thaliana*, grape (*Vitis vinifera*) and rice (*Oryza sativa*). By analysis of the phylogenetic relationship, intraspecies and interspecies differences in five plant species, gene duplication, origins and evolution were revealed. These results may contribute to the extrapolation of *GRF* gene function from one lineage to another.

## Materials and Methods

### Database Searches for Highly Conserved *GRF* Genes

In our study, the genomic data of pear, *Arabidopsis*, and *Oryza sativa*, were downloaded from the GigaDB database^1^, TAIR^2^, and the Rice Annotation Project^3^, respectively. And the genomic data of both poplar and grape were downloaded from the Phytozome database^4^. The WRC (PF08879.8) and QLQ (PF08880.9) domains were downloaded from the PFAM database^5^ (Punta et al., 2011) and were separately blasted against the corresponding plant genomes based on the hidden Markov model (HMM) using DNATools software. Subsequently, SMART software (Letunic et al., 2012) and the PFAM database (Punta et al., 2011) were used to identify the sequences that contain the WRC (Kim et al., 2003) and QLQ domains (Kim et al., 2003).

### Phylogenetic Analysis of *GRF* Genes

To construct the phylogeny of the *GRF* genes from the five flowering plant species, multiple sequence alignments for all amino acid sequences of the full-length GRF proteins were conducted using ClustalX software with the default settings. A phylogenetic tree was generated using the neighbor-joining (NJ) method using MEGA 7.0 software (Kumar et al., 2016) with the following parameters: pairwise deletion mode, Poisson correction, and bootstrapping (1000 replicates).

### Gene Structure Analysis and Motif Detection

The exon-intron structures of the *GRF* family were drawn using the GSDS website (Guo et al., 2007) (Gene Structure Display Server^6^) by comparing the coding sequences with their corresponding genomic sequences.

The conserved protein motifs were analyzed using the MEME online tool (Bailey et al., 2015) (Multiple Expectation Maximization for Motif Elicitation ^7^ with the following parameters: maximum number of motifs of 20 and the optimum width from 6 to 200 residues. Additionally, we used the Pfam (Punta et al., 2011) and SMART databases (Letunic et al., 2012) to annotate the structural motif. All of the *GRF* gene functional annotations were obtained from Gene Ontology (GO ^8^) using Blast2GO software (Conesa et al., 2005).

### Microsynteny Analysis

Microsynteny analysis of chromosome segments containing *GRF* genes can identify and classify the expansion pattern of the *GRF* gene family. The physical locations of all *GRF* members on the chromosomes from pear, *Populus*, grape, *Arabidopsis* and rice were determined. If more than one gene family member was located in the same or neighboring regions of the genome, they were thought to be tandem duplications. If the two *GRF* genes were located on duplication blocks and their flanking protein-coding genes were highly similar at the amino acid level (Maher et al., 2006), they were thought to be large-scale duplication events. First, all of the *GRF* genes were located in the genome as the initial anchor point. Then, the protein-coding sequences 100 kb upstream and downstream of each anchor point were analyzed using the BLASTP program (Deleu et al., 2007) to identify whether the duplicated genes existed between the two independent regions. Then, the number of protein-coding genes exhibiting the highest non-self match (*E*-value < 10-10) (Sato et al., 2008) between the two flanking sequences of the anchor points was calculated. When four or more gene pairs with the synteny relationship were detected between the two regions, then these two regions were thought to originate from large-scale duplication events.

### Environmental Selection Pressure and Duplication Event Dating Analysis

The Ks and the Ka/Ks ratios of gene pairs in the duplication blocks were calculated. The protein sequences of gene pairs were compared using MUSCLE software (Edgar, 2004). And results from protein sequence alignment were used to guide the comparison of nucleic acid sequence codons using the PAL2NAL program (Suyama et al., 2006). The comparison results of the codons were imported into DnaSP software (Librado and Rozas, 2009). Then, the Ka/Ks and Ks ratios were calculated. In addition, the parameters used in the sliding window analysis were as follows: window size 150 bp and step size 9 bp.

### Functional Divergence Analysis

The functional divergence (I type and II type) between each *GRF* subfamily was calculated using V3.0B1 DIVERGE (Gu et al., 2013) software in accordance with the constructed phylogenetic tree. Type I functional divergence occurred after gene duplication, which usually led to a selectivity change in a specific amino acid, i.e., change in the evolution rate. The coefficient *𝜃*_I_ fluctuates from 0 to 1, reflecting weak to strong functional divergence between gene categories. Type II functional divergence occurred after gene duplication but only resulted in a change in the physical and chemical properties of amino acids (Gu, 1999, 2006). The evolutionary rate difference coefficient *𝜃*_I_ of each GRF subfamily and amino acid physico-chemical properties of the divergence coefficient *𝜃*_II_ and the corresponding posterior probability (Qk) were obtained. If Qk > 0.9, it was inferred that the amino acid site should have a functional differentiation after gene duplication.

## Results

### Identification and Chromosomal Location of *GRF* Genes in Five Genomes

**FIGURE 1:**
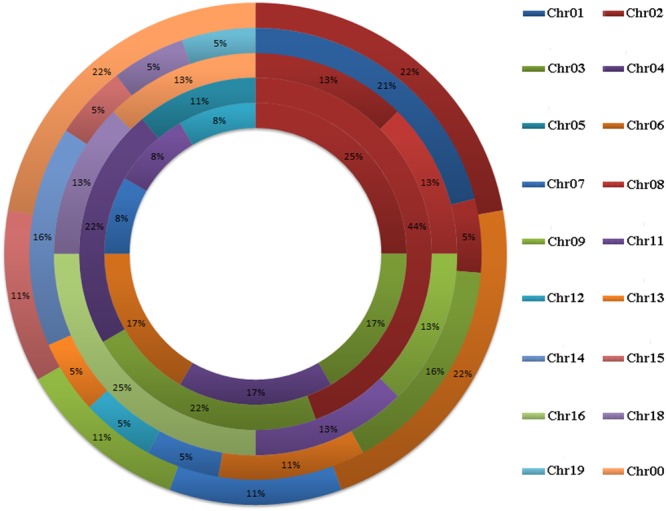
**Chromosomal distribution and percentage share of *GRF* genes in pear, *Populus, Arabidopsis*, grape and *Oryza sativa*.** The outermost ring represents chromosomes of pear, followed by *Populus*, grape, and *Arabidopsis*, and the innermost ring represents *Oryza sativa*.

A total of 58 *GRF* genes were identified from the five species studied, with 10 in pear (*PbGRF01* to *PbGRF10*), 19 in poplar (*PtGRF01* to *PtGRF19*), 9 in *Arabidopsis* (*AtGRF01* to *AtGRF09*), 8 in grape (*VvGRF01* to *VvGRF08*) and 12 in *Oryza sativa* (*OsGRF01* to *OsGRF12*), respectively. In addition, we determined the physical location of *GRF* genes on the chromosomes according to the overall search in the complete genome sequences of the five plant species (**Supplementary Table S1**). The results showed that the distribution of the 58 *GRF* genes among the chromosomes of the five species was not even (**Figure 1**). In the *Arabidopsis* and *Oryza sativa* genomes, the *GRF* genes were mainly distributed on chromosome 2, chromosome 3 (2) and chromosome 4 (2). For grape, *GRF* genes were found on chromosomes 2, 8, 9, 11, 16, and 18. In poplar, *GRF* genes were distributed on chromosomes 1, 2, 3, 6, 7, 12, 13, 14, 15, 18, and 19. In pear, we found *GRF* genes on chromosomes 2, 6, 7, 9, and 15. Additionally, three *GRF* genes (*VvGRF08, PbGRF08*, and *PbGRF09*) could not be mapped to any chromosome in the grape or pear genomes (**Supplementary Table S1**).

### Evolutionary Analysis of *GRF* Genes in Rice, Grape, *Arabidopsis, Populus*, and Pear

**FIGURE 2:**
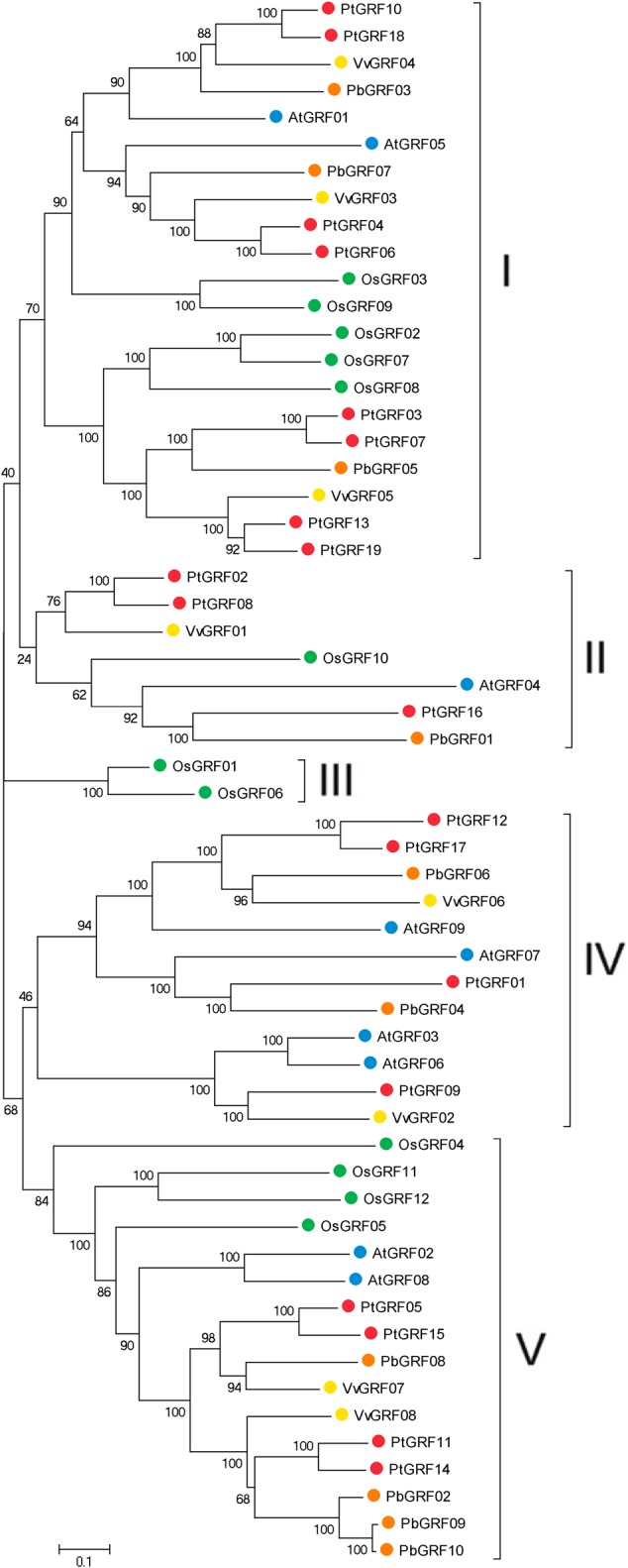
**Phylogenetic analysis of *GRF* genes in pear, *Populus, Arabidopsis*, grape and *Oryza sativa*.** The species background for each GRF protein is represented by different colors. Based on the bootstrap values and evolutionary distances, the tree was clustered into five subfamilies. Gene names are listed in **Supplementary Table S1**. The scale bar represents 0.1 amino acid changes per site.

Using the well-described GRF proteins in representative plant species, including the monocots *Brachypodium distachyon, Oryza sativa, Setaria italic, Zea mays, Sorghum bicolor* and the dicots *Arabidopsis thaliana, Populus trichocarpa, Glycine max, Citrus sinensis, Vitis vinifera, Cucumis sativus, Brassica rapa* and Chinese pear, the evolutionary relationships between members of the GRF families proteins were evaluated through phylogenetic analysis. According to the nodes of the phylogenetic tree, the NJ tree could be divided into five subfamilies, designated as I, II, III, IV, and V, respectively (**Supplementary Figure S1**). Subsequently, to further understand the similarity and evolutionary history of the *GRF* genes in rice, grape, *Arabidopsis, Populus* and Chinese pear, we built an unrooted phylogenetic tree using the NJ method in the MEGA7 software (Kumar et al., 2016). The NJ tree showed that 58 GRF proteins were divided into five subfamilies (**Figure 2**), which was consistent with the result from phylogenetic analysis (**Supplementary Figure S1**). The topology of these two phylogenetic trees and the distribution of GRF gene in each subfamily were basically the same (**Figure 2**; **Supplementary Figure S1**). Therefore, we focused our research on the evolution of the GRF family members in rice, grape, *Arabidopsis, Populus* and pear. As shown in **Figure 2**, subfamily III contained the minimal *GRF* numbers (2), and subfamily I has the maximal GRF numbers (21), followed by subfamily V (16) and subfamily IV (12). Each of the five species (rice, grape, *Arabidopsis, Populus* and pear) contributed at least one *GRF* gene to subfamily I, subfamily II and subfamily V, whereas, the members of subfamily III and subfamily IV included one, two or three species. Subfamily III consisted of only rice (monocots) and subfamily IV consisted of grape, *Arabidopsis, Populus*, and pear (dicots). Therefore, we deduced that this phenomenon may correspond to a special gene expansion event (lost or obtained) during the evolutionary process (**Supplementary Figure S1**; **Figure 2**). In addition, according to the phylogenetic tree (**Figure 2**), we identified pairs of orthologous genes among the *GRF* genes: *PbGRF01* and *PtGRF16*, and *PbGRF06* and *VvGRF06*, and *PbGRF04* and *PtGRF01*.

### Structural Analysis of *GRF* Genes from Pear, Poplar, Grape, *Arabidopsis* and Rice

**FIGURE 3:**
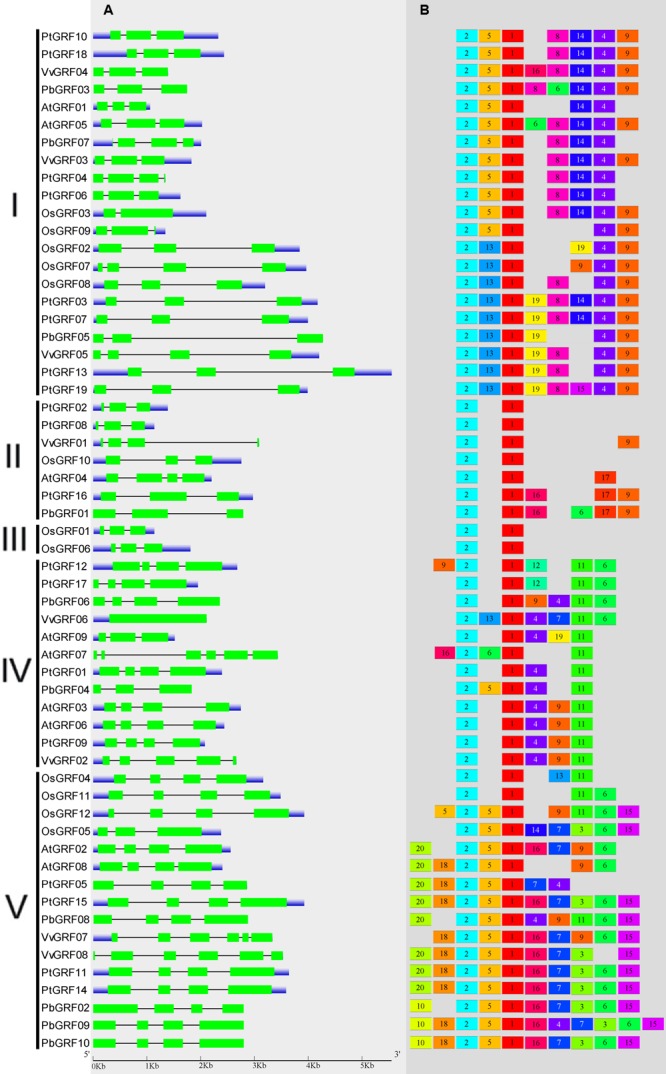
**Exon-intron structure and motif compositions of *GRF* genes across five plant species. (A)** Exon-intron structures of the *GRF* genes. Green rectangles: exons; thin lines: introns; blue boxes: untranslated regions (UTRs). **(B)** MEME motif search results. Note that different motifs are represented by different color boxes and that the length of each box does not show the true motif size.

To gain more insights into the structural diversity of *GRF* genes, exon-intron pattern maps of the individual *GRF* genes were generated. As shown in **Figure 3A**, the 58 *GRF* genes contained different numbers of exons, varying from 1 to 6. We found that 27 *GRF* genes had four introns and 25 had three exons, three genes had six introns and one had five exons, one gene had two exons, and *vGRF06* had only one exon. These results implied that both of exon gain and loss occurred during the evolution of *GRF* genes, which may help to explain the functional diversity of closely related *GRF* genes. Exon-intron structures of the paralogous and orthologous *GRF* gene pairs were further analyzed. Among these gene pairs, the exon number of five gene pairs exhibits exon-intron gain or loss variations, including *PtGRF04*/*PtGRF06, OsGRF02*/*OsGRF07, PbGRF06*/*VvGRF06, PtGRF01*/*PbGRF04*, and *PtGRF09*/*VvGRF02* (**Figure 3A**). In comparing the five gene pairs, *PtGRF06, OsGRF02, PbGRF04* and *PtGRF09* lost one exon whereas *PtGRF04, OsGRF07, PtGRF01* and *VvGRF02* gained one exon during the long evolutionary period. We speculated that these differences are possibly due to a single intron gain or loss event during the long evolutionary period.

**FIGURE 4:**
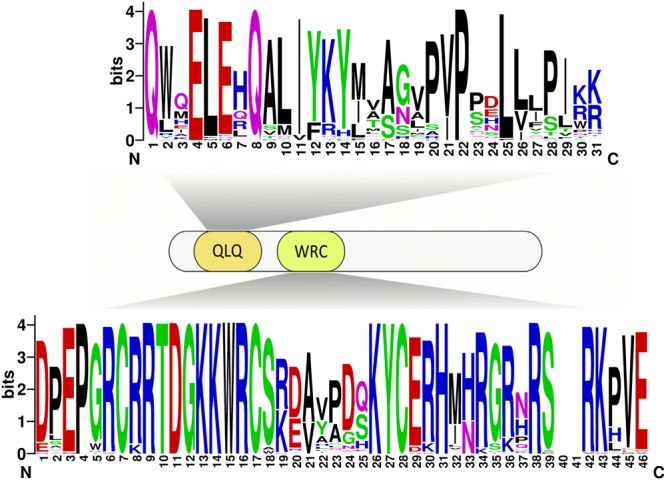
**Domain composition of GRFs.** Fifty-eight GRFs have both the characteristic WRC and QLQ domains. The sequences of the QLQ and WRC domains of the 58 GRFs were aligned using ClustalW software to analyze their sequence features. These domain diagrams were plotted using the online WebLogo tool.

Due to no high similarity among the 58 *GRF* genes, thus we used MEME software to identify the conserved motifs in the 58 GRF proteins. Twenty motifs were found on the GRF proteins (**Figure 3B**; **Supplementary Table S2**). Subsequently, the Pfam and SMART databases were used to annotate each of the putative motifs. Motif 1 and motif 17 were identified to encode the WRC domain (**Figure 4**), and motif 2 was found to encode the QLQ domain (**Figure 4**), while the remaining motifs did not have function annotation. As shown in **Figure 3B**, the most closely related members in each subfamily exhibit common motif compositions (e.g., PtGRF10 and PtGRF18, VvGRF05 and PtGRF13), implying functional similarities among GRF proteins. Both motif 1 and motif 2 were present in all 58 GRF proteins and thought as the most conserved motifs. In addition, some subfamily specific motifs, such as motif 18 and motif 20 in the subfamily V, were also found, implying that they might be important for the functions of GRF proteins in this subfamily. To further understand the function of different *GRF* genes, we searched the GO Database using Blast2GO software. The results show that the 58 *GRF* genes contain common functions such as regulation of metabolic process, biological process, organic cyclic compound binding, molecular function, intracellular organelles, and cellular components (**Supplementary Table S3**).

### Conserved Microsyntenies Were Found in Five Plant Species

**FIGURE 5:**
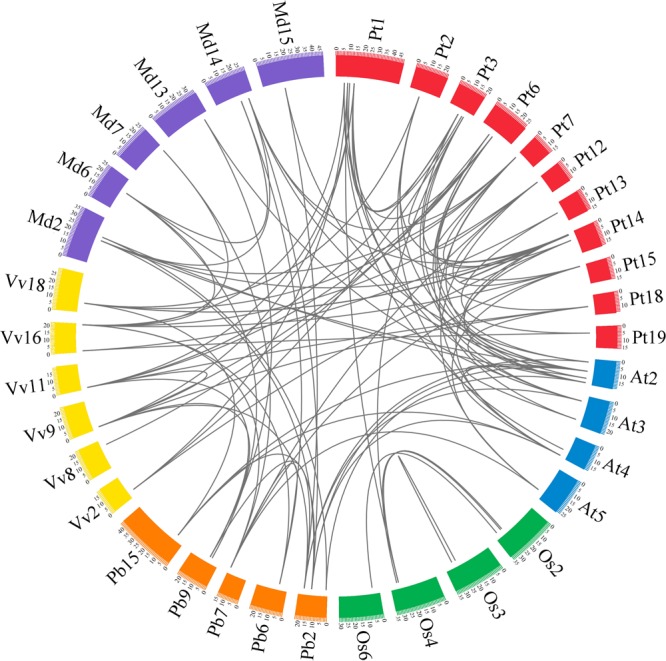
**Extensive microsynteny of GRF regions across six plant species.** The pear, apple, *Populus*, grape, *Arabidopsis* and rice chromosomes are depicted as different color boxes and labeled Pb, Md, Pt, Vv, At and Os, respectively. The scale on the circle is in megabases. Syntenic relationships between GRF regions are represented by black lines. The apple *GRF* gene family was obtained from The Apple Gene Function and Gene Family Database (Zhang et al., 2013).

In previous studies, microsynteny analysis of several plant species was performed to identify the location of homologous genes (Li et al., 2014; Lin et al., 2014; Wang et al., 2015; Cao et al., 2016b). In our research, microsynteny was investigated to infer the relationship of the *GRF* genes between eudicots (pear, *Populus*, grape and *Arabidopsis*) and monocots (rice). Additionally, since apple (Velasco et al., 2010) and pear (Wu et al., 2013) belong to the Rosaceae species, apple was also considered in the following analysis. The members of the *GRF* family of the six plant species (pear, apple, *Populus*, grape, *Arabidopsis* and rice) were used as anchor genes to clarify the molecular history of the surrounding chromosomal regions. Through pairwise comparisons and comparison of all of the proteins in the *GRF* gene flanking areas, the conserved microsyntenies were found in the six plant species (**Figure 5**).

Firstly, we identified intraspecies relationships of the *GRF* genes. A total of 14 collinear gene pairs in *Populus*, six in pear, four in apple, four in rice, two in *Arabidopsis*, and one in grape were found, respectively (**Figure 5**; **Supplementary Table S4**). Additionally, 15 *GRF* genes were not distributed in any microsynteny, such as *PbGRF05* in pear and *VvGRF01* in grape. These results revealed that there was not only a whole-genome duplication event but independent duplication events as well.

Subsequently, we analyzed the corresponding interspecies microsynteny in the six plant species. Twenty-five *GRF* genes were not present in the interspecies microsynteny analysis, including seven *OsGRFs*, six *VvGRFs*, five *AtGRFs*, three *PtGRFs*, two *PbGRFs* and two *MdGRFs*. By microsynteny analysis, 91 conserved syntenic segments were found (**Figure 5**; **Supplementary Table S4**). Among them, fourteen orthologous gene pairs were identified from pear and apple, 8 orthologous gene pairs were identified from pear and *Arabidopsis*, 7 orthologous gene pairs were identified from pear and grape, and 3 orthologous gene pairs were identified from pear and *Populus*. However, we did not find any orthologous gene pairs between pear and rice. These results may reflect that the relationship between pear and apple was closer than that between pear and grape/*Arabidopsis*/*Populus*/rice. Remarkably, some collinear gene pairs detected between pear and *Arabidopsis*/grape were not identified between pear and *Populus*, such as *PbGRF07*/*VvGRF03, PbGRF08*/*AtGRF02*, and *PbGRF07*/*AtGRF05* (**Figure 5**; **Supplementary Table S4**), suggesting that these orthologous pairs were generated after *Populus* diverged from the common ancestor of pear and grape/*Arabidopsis*. In addition, we observed that two or more *GRF* genes from apple, *Populus, Arabidopsi*s and grape matched one pear *GRF* gene (**Supplementary Table S4**), such as *VvGRF03* and *VvGRF04* orthologous to *PbGRF07* and *AtGRF02* and *AtGRF08* orthologous to *PbGRF08* (**Supplementary Table S4**), implying that these genes are probably paralogous gene pairs.

### Gene Duplication of *GRF* Genes

The *GRF* gene family may have experienced many duplication processes, including whole-genome duplication, segmental duplication and tandem duplication, during evolution (Moore and Purugganan, 2003). To further understand the evolution of *GRF* genes, the gene duplication events of the *GRF* family were identified in five plant species (pear, *Populus*, grape, *Arabidopsis* and rice). The similarity of *GRF* flanking sequences was searched. If four or more genes in the upstream and downstream 100 kb of the two corresponding *GRF* genes obtained the best non-self match using the BLASTP program (*E*-value < 10-10), then these two regions were thought as the result of a large-scale duplication event. To avoid the *GRF* gene pairs that were located in the duplication region with the larger genetic difference, a set of relaxed criteria for gene gathering was defined according to a pair of flanking sequences of the *GRF* gene containing two or three conserved genes.

**FIGURE 6:**
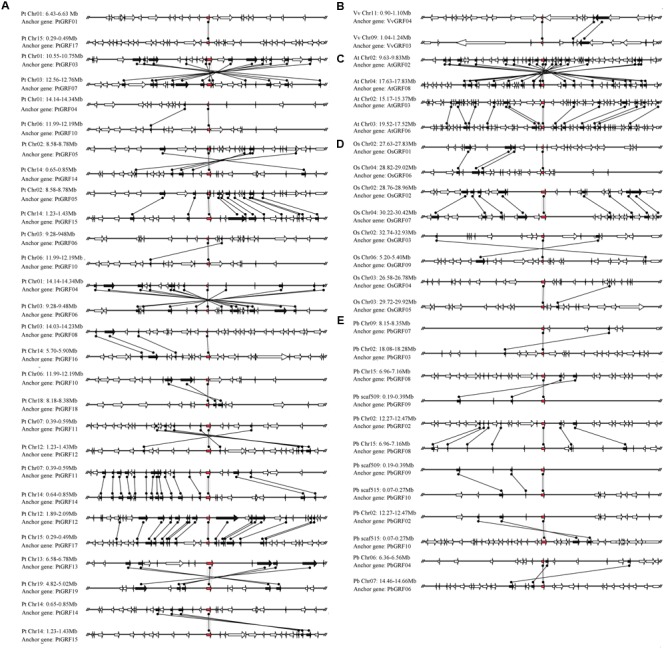
**Microsynteny maps of *GRF* genes in *Populus***(A)**,** grape **(B)**, *Arabidopsis* **(C),** rice **(D)** and pear **(E)**. The relative positions of all flanking protein-coding genes were defined by the anchored *GRF* genes, highlighted in red. Gray horizontal lines indicate the chromosome segments. Transcriptional orientations are represented by arrows. A gray line connects the conserved gene pairs among the segments.

The pear genome contained nine *GRF* genes, eight of which (approximately 88.9%) were found in the duplication region of the genome (**Figure 6E**). In these gene pairs, six conserved genes were found in the flanking sequences of *PbGRF02*/*PbGRF08*; thus, this pair of genes was thought to have evolved from a large-scale duplication event. Nineteen *GRF* genes were included in the *Populus* genome, and 17 of these genes (approximately 89.5%) were found to be distributed on the duplicated segments of chromosomes (**Figure 6A**). As these genes (*PtGRF03*/*PtGRF07, PtGRF05*/*PtGRF14, PtGRF05*/*PtGRF15, PtGRF04*/*PtGRF06, PtGRF11*/*PtGRF12, PtGRF11*/*PtGRF14, PtGRF12*/*PtGRF17, PtGRF13*/*PtGRF19*, PtGRF14/*PtGRF15, PtGRF08*/*PtGRF16*) were located in high synteny regions (**Figure 6A**), their pairs were speculated to evolve from large-scale duplication events. In addition, the gene pair of *PtGRF14* and *PtGRF15* were located in the adjacent positions of chromosome 14 (**Figure 6A**), and therefore might be produced by tandem duplication. The *Arabidopsis* genome contained 9 *GRF* genes, four of which were found in the duplication regions of the genome. The flanking sequences of two gene pairs (*AtGRF02*/*AtGRF08, AtGRF03*/*AtGRF0*6) had remarkable synteny (**Figure 6C**) and were inferred to have evolved from large-scale duplication events. Moreover, eight of 12 *GRF* genes were found in the duplication regions of the rice genome (**Figure 6D**). Conserved gene sequences were found in adjacent sequences of two gene pairs (*OsGRF01*/*OsGRF06* and *OsGRF02*/*OsGRF07*), indicating that they were evolved from large-scale duplication events. In contrast, as *OsGRF04* and *OsGRF05* were located in adjacent positions on the same chromosome, the gene pair should be produced by tandem duplication. Only two of 8 *GRF* genes were found in the duplication regions of the grape genome. The synteny of the gene pair *VvGRF03*/*VvGRF04* was weak in the duplicated region of the genome, and only two conserved genes were found on the flanking sequences (**Figure 6B**).

### Strong Purifying Selection for *GRF* Gene Pairs in Pear

To understand how gene duplications evolved into distinct *GRF* genes with different functions, we investigated the non-synonymous (Ka) and synonymous (Ks) substitutions and the ratio of Ka/Ks during the evolution of the *GRF* gene family in five plant species. In general, Ka/Ks < 1 indicates negative or purifying selection with functional constraint, Ka/Ks = 1 indicates neutral selection, and Ka/Ks > 1 indicates positive selection.

**FIGURE 7:**
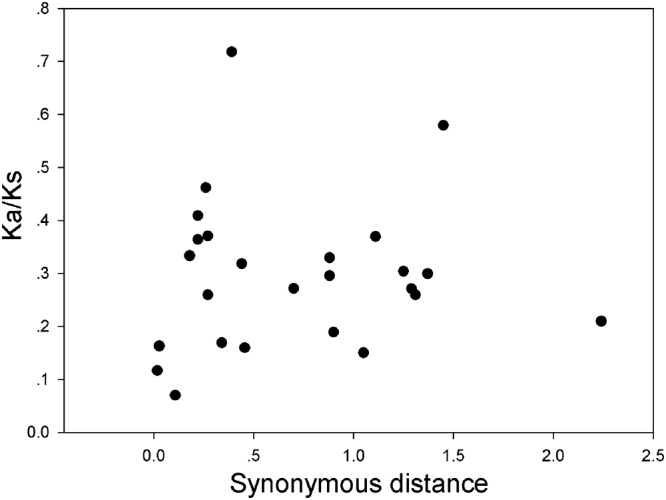
**Scatter plots of the Ka/Ks ratios of duplicated *GRF* genes in pear, *Populus*, grape, *Arabidopsis* and rice.** The *X*- and *Y*-axes denote the synonymous distance and Ka/Ks for each pair, respectively.

**FIGURE 8:**
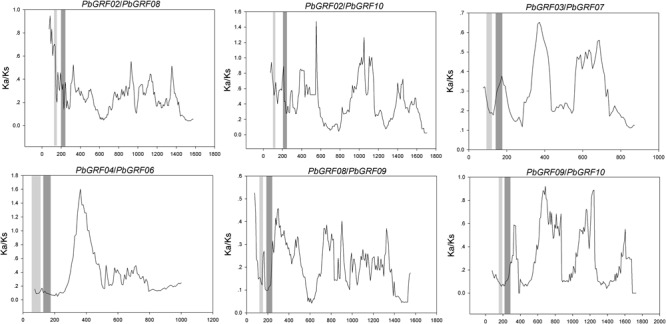
**Sliding window plots of duplicated *GRF* genes in pear.** The gray blocks, from light to dark, represent the positions of the WRC and QLQ domains, respectively. The window size is 150 bp, and the step size is 9 bp.

In our research, the Ka/Ks ratios of all pear *GRF* paralogous pairs were less than 0.2 (**Supplementary Table S5**), indicating that the *GRF* gene family evolved under strong purifying selection. Thus, we concluded that the *GRF* genes were slowly evolved at the protein level in pear. Remarkably, 27 *GRF* gene pairs appeared to be under purifying selection (**Figure 7**), because of their Ka/Ks ratios less than 1. Subsequently, we performed a sliding-window analysis of Ka/Ks between each pair of GRF paralogous and further clarified the selection pressures in pear. As shown in **Figure 8**, most Ka/Ks across coding regions were much less than one, with exception for one or several distinct peaks (Ka/Ks > 1). Compared with the other regions (peaks), the WRC and QLQ domains generally had lower Ka/Ks ratios (valleys), consistent with functional constraint being dominant in these domains. Together with the sliding window and Ka/Ks analysis (**Figure 8**), we deduced that strong purifying selections might have played a key role in the evolution of *GRF* genes, especially for the WRC and QLQ domains in pear.

### Functional Divergence in the *GRF* Gene Family

To understand whether amino acid substitutions in the *GRF* gene family caused adaptive functional diversification, DIVERGE 3.0B1 software (Gaucher et al., 2002; Gu et al., 2013) was used to estimate type I and type II functional divergence between gene subfamilies in the *GRF* gene family based on posterior analysis. In addition, because subfamily III contains only two sequences, which is less than 4 sequences required by the DIVERGE software analysis (Gaucher et al., 2002; Gu et al., 2013), we did not analyze subfamily III. Type I functional divergence usually results in a selective change in the specific amino acid, that is, the rate of evolution changes. The results revealed that *𝜃*_I_ was not gained between subfamily V and subfamily I or subfamily IV. This may be caused by an ML of less than 0 (**Table 1**). A card test (x 2) was performed for groups with a *𝜃*_I_. The *P*-values of groups I and II, I and IV, II and IV, II and V were less than 0.05, reaching a significant level. To avoid the occurrence of false positives, we determined the sites of posterior probability (Qk) > 0.9 to be key amino acid sites leading to functional divergence according to previous research methods (Yin et al., 2013). The results showed there were significant type I functional differences in the 295th amino acid between I and II, in the 231st and 326th amino acid sites between I and IV, and in the 295th amino acid site between II and IV.

**Table 1:** Analysis of type I functional divergence.

GRF subfamilies	𝜃_I_	𝜃_SE_^a^	𝜃_LRT_^b^	Q _k_ > 0.9	*P*
I vs. II	0.253346	0.098452	11.316795	295	*P* < 0.05
I vs. IV	0.204651	0.056893	11.579667	231, 326	*P* < 0.05
I vs. V	–	–	–	–	–
II vs. IV	0.207995	0.081973	7.413967	295	*P* < 0.05
II vs. V	0.202968	0.106781	4.688024	207	*P* < 0.05
IV vs. V	–	–	–	–	–

*^a^Standard error; ^b^Value of likelihood ratio test.*

Type II functional divergence occurred after gene duplication, which only resulted in the change in the physical and chemical properties of amino acids. As shown in **Table 2**, the type II functional divergence coefficients between any two subfamilies are relatively small, some even negative (groups for which *𝜃*_II_ is negative were not included in the detailed analysis). Subfamilies II and V had three key amino acid sites (207, 327, and 331), and subfamilies I and IV had a critical amino acid site (231) at a locus that is a key site in the type I functional divergence analysis, suggesting that this locus may have a very close relationship with the change in GRF function (**Table 2**). Three key amino acid sites (207, 327, and 331) were detected in both subfamilies II and V. And another key amino acid site (231) in the type I functional divergence analysis, was detected in both subfamilies I and IV, suggesting that this site may have a very close relationship with the change in *GRF* gene function.

**Table 2:** Analysis of type II functional divergence.

GRF subfamilies	𝜃_II_	𝜃_SE_^a^	Q_k_ > 0.9	No. of sites
I vs. II	-0.041743	0.177428	–	–
I vs. IV	0.019023	0.163690	231	1
I vs. V	-0.096491	0.145651	–	–
II vs. IV	-0.078060	0.189260	–	–
II vs. V	0.000900	0.165205	207, 327, 331	3
IV vs. V	-0.507273	0.211017	–	–

*^a^Standard error.*

## Discussion

Growth-regulating factors are plant-specific transcription factors that play key roles in plant growth and development. In our research, by searching local genome databases, 19, 12, 10, 9, and 8 *GRF* genes were identified in *Populus*, rice, pear, *Arabidopsis*, and grape, respectively. The *GRF* genes were divided into five classes, and orthologous pairs of pear and grape GRF proteins were more common according to the topology of phylogenetic tree, which revealed that some ancestor *GRF* genes existed before the divergence of pear and grape during evolution.

There exist functional differences of GRFs between the five plant species, which might be related to the diversity both of *GRF* genes’ exon-intron structures and motif components. In our study, the 58 *GRF* genes contain different numbers of introns/exons, implying that there is diversity in the *GRF* genes of the five plant species. For example, the *GRF* gene *AtGRF07* contains five exons, while the *VvGRF06* has only one exon. Nevertheless, the most closely related *GRF* genes shared similar exon-intron structure and motif composition in the same subfamily, either in their exon lengths or intron numbers. Furthermore, different conserved protein motifs were present in individual GRF proteins based on the MEME analysis. The differences in these features among the subfamilies revealed that the GRF members were functionally diversified. Interestingly, all known GRF proteins have motif 1 and motif 2, which encode a conserved WRC domain (containing a Trp-Arg-Cys structure) (Kim et al., 2003) and QLQ domain (containing a Gln, Leu, Gln structure) (Kim et al., 2003), respectively. Among these domains, the WRC domain is known as the zinc-finger structure (Rushton et al., 1995). As shown in **Figure 4** and **Supplementary Figure S2**, zinc-finger structures are tightly connected in WRC motifs, implying that this domain functions in DNA binding.

Based on the comparative genome analyses, although the chromosome numbers and genome sizes of different plant species were diverse, gene orders among the related species were still highly conserved in the process of million years of evolution (Devos and Gale, 2000). Comparisons among the *GRF* genes across the five plant species’ genomic sequences implied the presence of one or more large-scale genome duplications during early evolution. Strong microsynteny was detected in the five dicot (pear, apple, *Populus, Arabidopsis*, and grape) genomes. In contrast, little or no microsynteny was detected between the five dicots (pear, apple, *Populus, Arabidopsis*, and grape) and one monocot (rice). For example, the low microsynteny (two pairs) of *GRF* genes from five dicots (pear, apple, *Populus*, grape, and *Arabidopsis*) and a monocot (rice) was probably because these plants are not closely related. Remarkably, the synteny blocks (14) in the *Populus* genome were much higher than the synteny blocks of the monocot (rice) and four other dicot (pear, apple, grape, and *Arabidopsis*) genomes, revealing that *Populus GRF* genes might have undergone large-scale duplication events during evolution, as shown in **Figure 6A**. Interestingly, we did not observe microsynteny relationships among *OsGRF01*-*05, OsGRF07*-*12, PtGRF03, PtGRF07, PbGRF09, PbGRF10, MdGRF03, VvGRF03* and *VvGRF04* and other 18 *GRF* gene members in these genomes studied, implying that these genes were either formed through complete transposition and loss of their primogenitors or ancient ones without detectable linkage to other *GRF* genes.

Gene duplications are of the major driving forces for generating novel genes, which would help organisms adapt to complex environments. Both events of tandem duplication and large-scale duplication are the main patterns of gene family expansion in plants, such as the *MYB* gene family in pear (Cao et al., 2016a), *CHS* gene family in maize (Han et al., 2016), or *MYB* gene family in *Setaria italica* (Muthamilarasan et al., 2014). In the present study, a high frequency of *GRF* genes was distributed in duplicated blocks, implying that large-scale duplications (whole-genome or segmental duplication) contributed to the expansion of the *GRF* gene family in plants. The Ka/Ks of the 27 paralogous gene pairs suggest that purifying selection may be largely responsible for maintaining the functions of GRF proteins from the four dicots (pear, *Populus, Arabidopsis*, and grape) and one monocot (rice). Furthermore, the Ka/Ks of pear *GRF* paralogous gene pairs were less than 0.2, suggesting that these genes underwent slow evolutionary non-diversification following duplication. In addition, we detected strong positive selection in coding regions in several pear *GRF* gene pairs, implying functional differentiation.

We used DIVERGE software to analyze subfamily I and subfamily IV. In the GRF sequence analysis, we detected 231 key functional divergences in sites, and the analysis of type I and type II functional divergence assayed important amino acid sites that may lead to functional differentiation of GRF decisive sites; thus, our study provides a reference for subsequent researchers exploring GRF functional divergence.

## Conclusion

In the present study, 58 *GRF* gene members were analyzed, including their physical location, phylogenetic relationship, conserved microsynteny, gene duplication and Ka/Ks. By phylogenetic analysis, these *GRF* genes were divided into five subfamilies. In each subfamily, we found that gene structure and motif distribution features were relatively conserved. Based on genome sequences of the five species (pear, *Populus*, grape, *Arabidopsis*, and rice), a comprehensive analysis of *GRF* genes was performed and the results showed a wide range of synteny and the presence of one or more large-scale genome duplications during early evolution. Our results suggest that large-scale gene duplication was the major pattern of expansion for the vast majority of *GRF* genes. These genes were under strongly purifying selection and maintained their functional stability. The systematic analysis might contribute to the extrapolation of *GRF* gene function from one lineage to another.

## Author Contributions

YuC conceived of and designed the experiments; YuC and YH performed the experiments; YuC analyzed the data; YuC, YH, QJ, YL, and YoC contributed reagents/materials/analysis tools; YuC and YaH wrote the paper.

## Conflict of Interest Statement

The authors declare that the research was conducted in the absence of any commercial or financial relationships that could be construed as a potential conflict of interest.

## Footnotes

1 http://gigadb.org/site/index

2 http://www.arabidopsis.org/

3 http://rapdb.dna.affrc.go.jp/

4 http://phytozome.jgi.doe.gov/pz/portal.html

5 http://pfam.xfam.org/

6 http://gsds.cbi.pku.edu.cn/

7 http://alternate.meme-suite.org/

8 http://www.geneontology.org

## Supplementary Material

FIGURE S1 **Phylogenetic tree of the GRF proteins in monocots *Brachypodium distachyon, Oryza sativa, Setaria italic, Zea mays, Sorghum bicolor* and the dicots *Arabidopsis thaliana, Populus trichocarpa, Glycine max, Citrus sinensis, Vitis vinifera, Cucumis sativus, Brassica rapa* and Chinese pear.** For the thirteen-species GRF gene tree, the GRF proteins of *Brachypodium distachyon, Setaria italic, Zea mays, Sorghum bicolor, Glycine max, Citrus sinensis, Cucumis sativus* and *Brassica rapa* were obtained from PLAZA 3.0 database, The neighbor joining tree was constructed using MEGA7 software.

FIGURE S2 **Sequence alignment of GRF domains in GRF proteins from pear, *Populus*, grape, *Arabidopsis* and rice.** The locations of the WRC and QLQ domains are represented by black dots.

TABLE S1 **Annotation of *GRF* genes in pear, *Populus*, grape, *Arabidopsis* and rice**.

TABLE S2 **MEME motif sequences in GRF proteins**.

TABLE S3 **Blast2GO annotation details of 68 GRF protein sequences**.

TABLE S4 **Synteny data in pear, apple, *Populus*, grape, *Arabidopsis*, and rice.** The apple *GRF* gene family was obtained from The Apple Gene Function and Gene Family Database (Zhang et al., 2013).

TABLE S5 **Estimates of the dates for the large-scale duplication events in pear**.
