# Supplementary figures and images for: Comparative Genomic Analysis of the GRF Genes in Chinese Pear (Pyrus bretschneideri Rehd), Poplar (Populous), Grape (Vitis vinifera), Arabidopsis and Rice (Oryza sativa)
Source: Front Plant Sci. 2016 Nov 24;7:1750. doi: 10.3389/fpls.2016.01750 (PMC5121280; doi:10.3389/fpls.2016.01750)

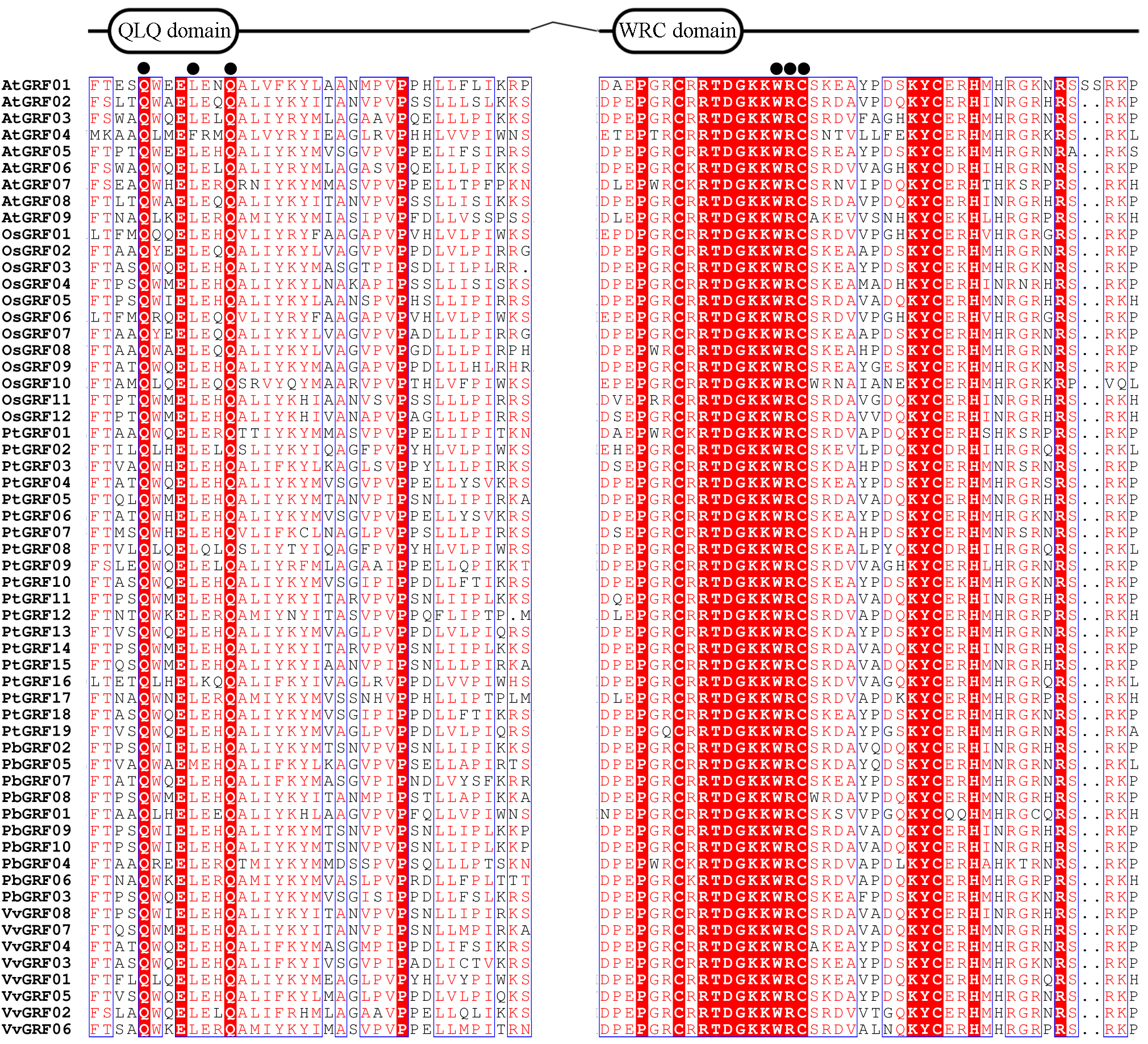

Supplement: FIGURE S2 — Sequence alignment of GRF domains in GRF proteins from pear, Populus, grape, Arabidopsis and rice. The locations of the WRC and QLQ domains are represented by black dots. [file Image_2.TIF]
